# Dynamic Response and Damage Accumulation of Laminated Composites under Repeated Low-Velocity Impacts

**DOI:** 10.3390/ma16020778

**Published:** 2023-01-12

**Authors:** Jin Sun, Linhai Huang, Yunfeng Dai

**Affiliations:** Jiangsu Key Laboratory of Advanced Food Manufacturing Equipment and Technology, Jiangnan University, Wuxi 214122, China

**Keywords:** repeated impacts, composite laminates, mechanical response, damage accumulation

## Abstract

The mechanical response and damage accumulation of carbon-fiber-reinforced composite laminates subjected to repeated low-velocity impacts were experimentally investigated. The repeated impact tests were conducted on [90_2_/−45_2_/0_2_/45_2_]_S_ quasi-isotropic and [90_2_/0_2_]_2S_ cross-ply composite laminates under 16.8 J impact energy, respectively. For each impact, impact responses such as force-time, force-displacement and energy-time curves were recorded. The trends of peak force, maximum central displacement, energy absorption rate and bending stiffness with the increasing impact number were summarized, and the maximum number of repeated impacts corresponded to the occurrence of penetration events. The results showed that the delamination initiation, fiber breakage and penetration were the three typical characteristics describing the damage evolution of the repeated impacts. The damage accumulation of both the laminates was characterized by employing appropriate damage indices. By contrast, the quasi-isotropic laminates had higher impact resistance and damage tolerance, and their damage accumulation was relatively slower.

## 1. Introduction

Fiber-reinforced composites have been widely used in aerospace, automotive, defense and energy industries due to their lightweight, high specific strength and high specific stiffness [1,2,3]. The composites in service are likely to be exposed to repeated impact loads such as hail impact and the impact of stones thrown by the tires of a plane during landing [4,5]. Although a single low-velocity impact may not externally damage the material, the long-term effect of repeated impacts will accumulate the damage, which can degrade the material performance and even lead to the failure of an apparently undamaged structure [6,7,8,9]. Therefore, the repeated low-velocity impact behaviors of composite structures have attracted more and more attention.

Experimental and numerical research has been conducted to study the repeated low-velocity impact responses of composites, which can be affected by many factors including impact energy, stacking sequence, specimen thickness, impactor shape, ambient temperature, etc. The dynamic responses of braided textile-reinforced composites under repeated low-velocity impacts studied by Wang et al. [10] show two types according to the level of normalized impact energy. Under low-impact energy, the initial micro-cracks appear in the yarns first, causing the dominant failure mode of delamination with a low damage accumulation. Referring to higher impact energy, matrix cracking is the first damage mode, followed by rapid delamination propagation, and the accumulated damage leads to the fast propagation of macro cracks and fiber ruptures. The studies by Sevkat et al. [11] reported that the repeated low-velocity impact responses of hybrid plain-woven composites including non-hybrid S2-glass-fiber (GL)/toughened epoxy and IM7 graphite fiber (GR)/toughened epoxy as well as hybrid S2-glass-IM7 graphite fiber/toughened epoxy composite panels are significantly influenced by the lay-up sequence. Under the premise of using the same amounts of glass and graphite fabrics, the hybrid GL/GR/GL composite survives twice the number of impacts compared with the hybrid GR/GL/GR composite, which suggests that using tougher material for the skin layers may delay the damage evolution. Atas et al. [12] demonstrated the thickness effect on the repeated impact response of woven E-glass/epoxy composites. The penetration threshold/energy for single-impact linearly changes with the thickness for the chosen composite plates. For the given thicknesses/layer numbers, the relationship between the impact energy and the impact number until complete penetration of the specimens can be described by particular equations using power regression methods, which is well suited for predicting the impact number until penetration under relatively small impact energies without testing. Zhou et al. [13] also paid attention to the thickness effect when numerically studying the dynamic mechanical response and damage mechanism of composite laminates subjected to repeated low-velocity impact. The impact-response parameters varied linearly with thickness, and the dominant damage mode changed from intra-laminar damage to inter-laminar damage with growing thickness due to the better carrying capacity of the thicker layer. Single and repeated low-velocity impact tests conducted by Dogan [14] showed that under the same impact energy, thermoplastic matrix-based composites have a higher penetration threshold than thermoset matrix-based composites. In addition, the threshold value is remarkably affected by the impactor shape. The conical impactor possesses a significantly lower perforation threshold than the hemispherical impactor. Sun et al. [15] experimentally and numerically explored the impact response, damage accumulation and energy dissipation distributions of patch-repaired carbon fiber reinforced polymer (CFRP) laminates under repeated impacts. The sudden drop of the force–time curve was an important signal of the serious internal damage of the patch, and the delamination damage can be regarded as a factor to show the damage accumulation degree of patch-repaired laminates. Icten [16] performed experiments to study the response of woven glass-epoxy composites to repeated impacts at room temperature and −50 °C. The impact number for perforation highly depended on the ambient temperature. For some cases, the impact number of the composite impacted at −50 °C was five times higher than that impacted at room temperature. The total failure area for perforation at −50 °C was larger than that at room temperature for the same repeated impact energy levels. Liao et al. [17] investigated the repeated low-velocity impact responses and damage accumulation mechanism of the composite laminates by considering the influence of four different impactor diameters. They proposed a new damage index, DI-B, based on bending stiffness reduction rate and normalized maximum displacement, which could characterize the damage accumulation of the laminates and distinguish the occurrence of penetration.

In practice, both the quasi-isotropic and orthogonal stacking patterns are typical and common for the laminated composites, and the corresponding impact resistance and damage tolerance are important factors to consider when choosing an appropriate stacking pattern under continuous impacts—which, however, has rarely been reported. In this paper, the repeated low-velocity impact behaviors of composite laminates with these two stacking patterns were studied. The repeated impact tests were performed until the penetration events occurred. The dynamic mechanical responses, including force-time, force-displacement and energy-time curves, were recorded. The tendencies of peak force, maximum central displacement, energy absorption rate and bending stiffness with the growing impact number were obtained, and the delamination initiation, fiber breakage and penetration were analyzed to describe the damage histories during the whole procedure of repeated impacts. In addition, specific damage indices were employed to characterize the damage accumulation of the laminates. Through the above analysis, the impact resistance and damage tolerance of the two structures were compared and estimated.

## 2. Experiment

### 2.1. Materials

The composite laminates comprising carbon fiber T300 as reinforced fiber and epoxy resin YH69 as the matrix were employed in this work. Table 1 shows the mechanical properties for each unidirectional laminate. The quasi-isotropic and orthogonal stacking patterns, namely [90_2_/−45_2_/0_2_/45_2_]_S_ and [90_2_/0_2_]_2S_, were adopted, respectively. Each ply had a thickness of 0.1 mm, and the size of the specimen was 150 mm × 100 mm.

### 2.2. Repeated Low-Velocity Impact Tests

**Figure 1:**
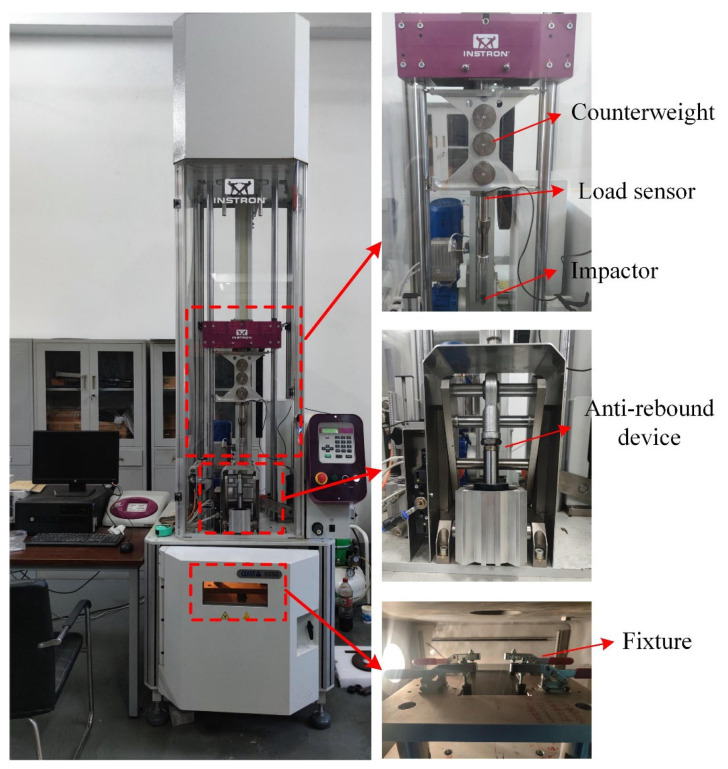
Testing machine for low-velocity impact.

The low-velocity impact tests were carried out using the Instron Dynatup 9350 HV drop-weight testing machine, as shown in Figure 1. A hemispherical shape impactor with a diameter of 16 mm was adopted. The four corners of the specimen were attached to the fixture with a hollow region by rubber clamping tweezers, and a free impact area of 125 mm × 75 mm was formed, leaving a constant impact point located in the center. During the repeated impact tests, the total mass of the drop hammer was 4.5 kg, and the impact velocity was 2.732 m/s. Successive single impacts with 16.8 J impact energy were imposed on the specimen until penetration occurred.

## 3. Results and Discussion

### 3.1. Dynamic Mechanical Response

**Figure 2:**
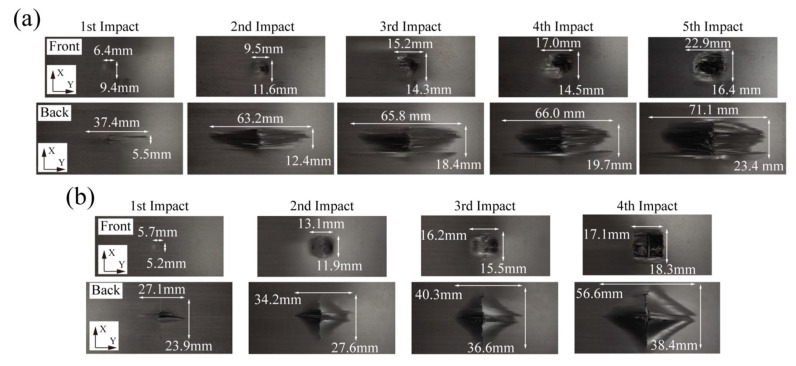
Damage morphology of laminates with stacking patterns of (**a**) [90_2_/−45_2_/0_2_/45_2_]_s_ and (**b**) [90_2_/0_2_]_2s_.

The surface damage morphologies of the laminates with two stacking patterns are demonstrated in Figure 2, where the *x* axis is along the length direction of the specimen, and the *y* axis the width direction. For both the laminates, the delamination appeared at the first impact, and a large number of fibers began to fracture at the second impact. Then, the damage continuously intensified until penetration occurred. During the repeated impact tests, the front sides of both the laminates were left with circular dents. The damage on the back side of the quasi-isotropic laminates extended along the stacking direction of outermost layer, while the damage on the back side of cross-ply laminates showed a cruciform distribution that was consistent with the two orthogonal stacking directions. The penetration of the two laminates appeared at the fifth and fourth impacts, respectively.

**Figure 3:**
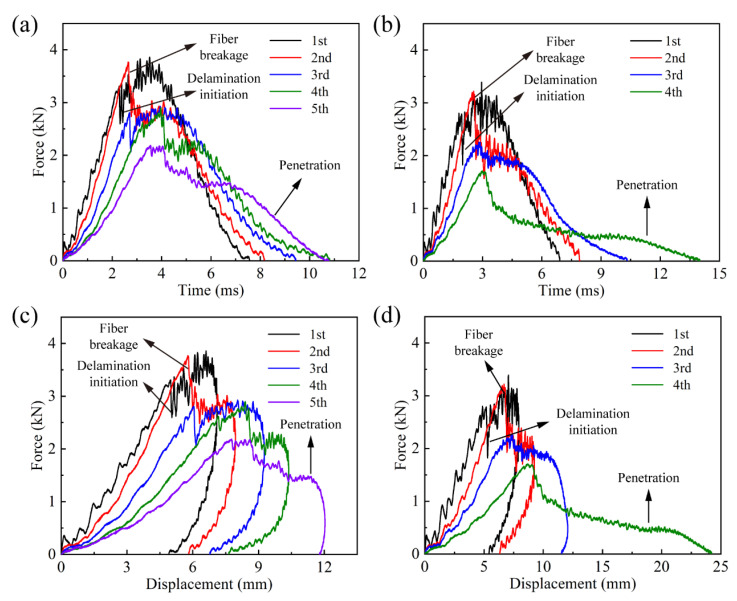
Force–time and force–displacement curves: (**a**) FT-[90_2_/-45_2_/0_2_/45_2_]_s_, (**b**) FT-[90_2_/0_2_]_2s_, (**c**) FD-[90_2_/-45_2_/0_2_/45_2_]_s_ and (**d**) FD-[90_2_/0_2_]_2s_.

Figure 3a,b show the force–time curves for the two laminates. The fluctuations of both the curves were significant at the first impact, and then gradually weakened as the impact number increased; hence, the curves became smoother. This is because more impact kinetic energy was absorbed due to the intensifying impact damage, while they could have been converted into the vibrations of the laminates before the occurrence and development of the damage. At the first impact, both curves grew linearly on the whole until the initial delamination appeared. After an obvious fluctuation, they continued to go up. As the repeated impacts proceeded, both curves showed a large decline at the second impact, which was due to the fact that a large amount of fibers began to fracture. Then, the curves produced more or less plateau at the fifth and fourth impact for the quasi-isotropic and cross-ply laminates, respectively. The occurrence of a plateau indicates the penetration events, since during penetration the impactor is embedded into the laminates and is required to overcome the friction between the impactor and the laminate, which keeps the impact force on a relatively stable value. In any case, the impact energy at penetration will be completely dissipated. By comparison, the quasi-isotropic laminates bore higher impact force to produce initial delamination and fiber breakage at corresponding impacts, and experienced more impacts until penetration.

Figure 3c,d depict the impact force–displacement curves of both the laminates. The delamination initiation, fiber breakage and penetration can also be reflected at corresponding impacts. The unrecoverable deformation of the two laminates was very close at the first two impacts, while that of the cross-ply laminates increased significantly after fiber breakage occurred, which indicated that the damage of this laminate caused by fiber breakage developed much more severely.

**Figure 4:**
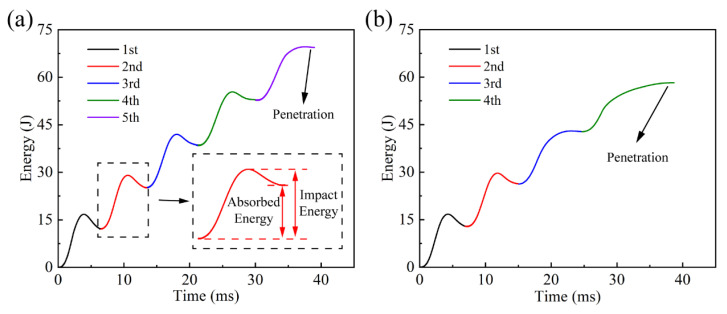
Energy–time curves for laminates with stacking patterns of (**a**) [90_2_/−45_2_/0_2_/45_2_]_s_ and (**b**) [90_2_/0_2_]_2s_.

Figure 4 shows energy–time curves covering all the repeated impacts of the two laminates. During each impact, part of the impact kinetic energy was transformed into the elastic energy of the specimen through contact deformation, while the other was absorbed by the damage, friction and vibration of the laminate. To some extent, the amount of energy absorption reflects how much damage accumulates, which means more energy absorption indicates more damage is produced. Under the same impact number, the absorbed energy of the quasi-isotropic laminates was less than that of the cross-ply ones, which indicated that the former stacking pattern had better rebounding performance and brought weaker damage at each impact. On the other hand, the total energy absorption of the quasi-isotropic laminates was relatively higher, which indicated that they accumulated more damage until penetration. On the whole, the quasi-isotropic laminates possessed higher impact resistance and damage tolerance.

**Figure 5:**
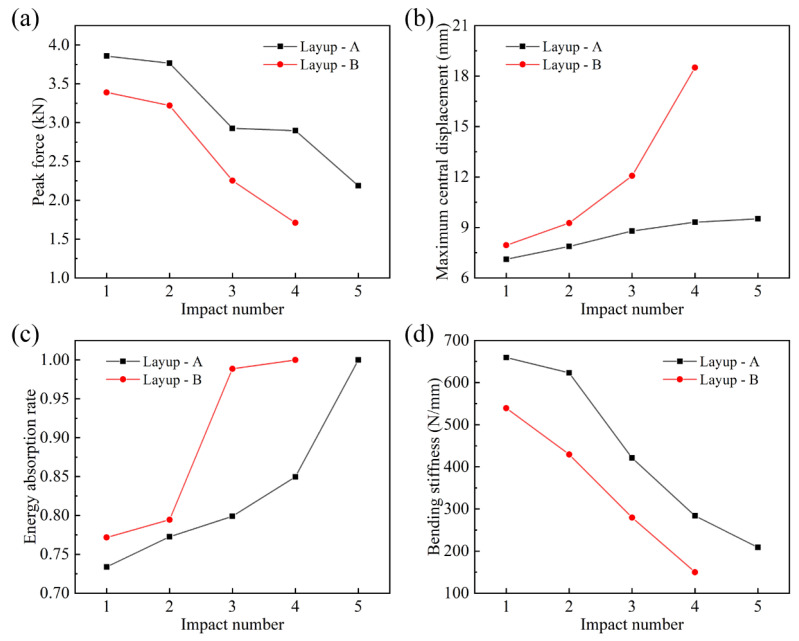
Mechanical characteristics of laminates with layup A ([90_2_/−45_2_/0_2_/45_2_]_s_) and layup B ([90_2_/0_2_]_2s_): (**a**) peak force–impact number curves, (**b**) maximum central displacement–impact number curves, (**c**) energy absorption rate–impact number curves and (**d**) bending stiffness–impact number curves.

The variations of peak force, maximum central displacement, energy absorption rate and bending stiffness with the impact number are described in Figure 5. As the impact number increased, the peak force of both the laminates declined continuously. Especially at the third impact, the decline was significant due to the appearance of fiber breakage at the second impact. The peak force of the quasi-isotropic laminates was always higher than that of the cross-ply laminates at the same impact, which indicated that the former laminates possessed a relatively higher load capacity. The maximum central displacement of the two laminates showed continuous growth with the impact number. The uptrend of curve A was relatively slow and stable, while curve B showed an accelerated rise, especially after the occurrence of fiber breakage. In addition, the maximum central displacement of the cross-ply laminates always remained larger than that of the quasi-isotropic ones. For both laminates, the energy absorption rate gradually went up as the impact number increased, which indicated that the impact damage intensified faster and faster, and the impact energy was absolutely absorbed at penetration. By contrast, the cross-ply laminates were affected more seriously by the occurrence of fiber breakage at the second impact, which greatly intensified their subsequent damage. The energy absorption rate of the quasi-isotropic laminates increased significantly at the last impact due to penetration. The bending stiffness of both the laminates, calculated by the slope of the ascending section in the impact force–displacement curves, declined with the increasing impact number; the stiffness of the quasi-isotropic laminates was always larger than that of the cross-ply ones, which indicated that the former stacking pattern could maintain the mechanical properties relatively better when suffering continuous impacts. For both laminates, the bending stiffness began to decrease due to the appearance and development of delamination damage after the first impact. Then, the impact-induced fiber breakage at the second impact caused the bending stiffness to more significantly drop during the following impacts. As the impact damage intensified, the bending stiffness continuously decreased at subsequent impacts until penetration.

### 3.2. Damage Accumulation Assessment

The damage evolution of laminates subjected to repeated impacts is a cumulative process from no damage to penetration; hence, it is important to characterize the damage accumulation of the laminates to evaluate their damage degree. In this paper, the damage variables DI and DI-B were introduced to assess the damage accumulation of the studied laminates, respectively.

**Figure 6:**
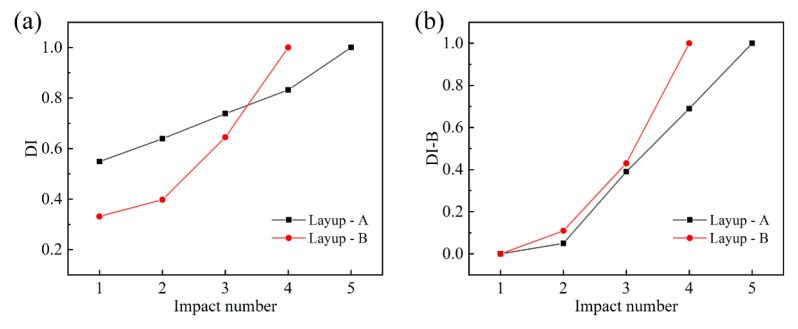
Damage accumulation of laminates with layup A ([90_2_/−45_2_/0_2_/45_2_]_s_) and layup B ([90_2_/0_2_]_2s_) characterized by (**a**) DI and (**b**) DI-B.

To study the damage accumulation in thick laminates from first impact to penetration, Belingardi et al. [18] developed the damage variable DI, expressed as follows:

$$\mathrm{DI}=\mathrm{DD}\frac{d_{max}}{d_{penetration}}$$

where *d_max_* is the maximum displacement in the impact force–displacement curves, and *d_penetration_* is the maximum displacement in the impact force–displacement curves at penetration. Before the appearance of penetration, *DD* is defined as

$$\mathrm{DD}=\frac{E_{a}}{E_{i}}$$

in which *E_a_* is the absorbed energy, and *E_i_* is the impact energy. After the penetration occurs, the value of DD is equal to one. Figure 6a shows the DI–impact number curves of the two laminates. The DI value of the cross-ply laminates increased faster than that of the quasi-isotropic laminates. The damage variable DI could describe the trend of energy absorption with the growth of the impact number, and the penetration could be easily identified. However, DI sometimes fails to characterize damage accumulation due to its non-monotonicity and non-zero initial value, since the value of DD at each impact is independent but not cumulative, which does not guarantee its monotonicity starting from zero.

To characterize the damage accumulation of composite laminates more generally, Liao et al. [14] proposed a damage variable, DI-B, based on bending stiffness reduction rate and normalized maximum displacement, expressed as follows:

$$\mathrm{DI}-B=R_{stiffness}\frac{d_{max}}{d_{penetration}}$$

where *R_stiffness_* is the bending stiffness reduction ratio, calculated by

$$R_{stiffness}=\frac{k_{0}-k_{i}}{k_{0}-k_{f}}$$

in which *k*_0_ is the initial bending stiffness at the first impact, *k_f_* is the bending stiffness at penetration, and *k_i_* is the bending stiffness at the *i*th impact. As the impact number increased, both the bending stiffness reduction rate and normalized maximum displacement grew monotonically from zero to one, which guaranteed that the value of DI-B was monotonic and increased from zero to one. Figure 6b describes the trend of DI-B with the impact number for both laminates. The DI-B value of the cross-ply laminates increased faster and was always larger than that of the quasi-isotropic ones, which indicated that the damage accumulation of the latter structures was relatively slower. The initial delamination at the first impact brought the two laminates relatively weak damage, and then the occurrence of fiber breakage at the second impact accelerated both their subsequent damage accumulation. Finally, the appearance of penetration could be identified when the DI-B reached the value of one.

## 4. Conclusions

The repeated impact tests were performed on T300/YH69 composite laminates with a quasi-isotropic/orthogonal stacking pattern to explore their dynamic response and damage accumulation under continuous low-velocity impacts. The impact resistance and damage tolerance of these two typical structures were compared and evaluated. The main conclusions are summarized as follows:

1. According to the force–time and force–displacement curves, the delamination initiation, fiber breakage and penetration were the three typical characteristics describing the damage evolution of repeated impacts. Compared with the cross-ply laminates, the quasi-isotropic laminates bore higher impact force to produce initial delamination/fiber breakage at the first/second impact and suffered more impacts until penetration.
2. The energy absorption of both laminates accumulated with the increasing impact number until penetration, and the impact energy at penetration was completely dissipated. The quasi-isotropic laminates absorbed less energy at each impact, while their total energy absorption was relatively higher, which reflected that this laminated structure possessed higher impact resistance and damage tolerance.
3. With the growth of the impact number, the peak force and bending stiffness of both laminates declined continuously, while their maximum central displacement and energy absorption rate increased. The occurrence of fiber breakage intensified these trends to varying degrees. By contrast, the quasi-isotropic laminates showed relatively higher carrying capacity and kept the mechanical properties relatively better when suffering continuous impacts.
4. Both the damage indices DI and DI-B showed that the damage accumulation of the quasi-isotropic laminates was relatively slower. By comparison, the index DI-B can characterize damage accumulation from no damage to penetration, corresponding to the value from zero to one, which is more consistent with the common definition of a damage variable.

## Figures and Tables

**Table 1:** Mechanical properties of T300/YH69 unidirectional laminate.

Mechanical Properties	Values
Longitudinal elastic modulus (*E*_11_)	130 GPa
Transverse elastic modulus (*E*_22_)	7 GPa
Shear modulus (*G*_12_)	3.6 GPa
Poisson’s ratio (*m*_12_)	0.3
Longitudinal tensile strength (*X^T^*)	1760 MPa
Longitudinal compressive strength (*X^C^*)	1100 MPa
Transverse tensile strength (*Y^T^*)	51 MPa
Transverse compressive strength (*Y^C^*)	167 MPa
Shear strength (*t*_12_)	70 MPa
Density (*r*)	1600 kg/m^3^

## References

[1] Boukar A., Corn S., Slangen P., Ienny P. (2022). Finite element modelling of low velocity impact test applied to biaxial glass fiber reinforced laminate composites. Int. J. Impact Eng..

[2] Ge X., Zhang P., Zhao F., Liu M., Liu J., Cheng Y. (2022). Experimental and numerical investigations on the dynamic response of woven carbon fiber reinforced thick composite laminates under low-velocity impact. Compos. Struct..

[3] Banik A., Zhang C., Khan M., Wilson M., Tan K. (2022). Low-velocity ice impact response and damage phenomena on steel and CFRP sandwich composite. Int. J. Impact Eng..

[4] Morais W.A., Monteiro S., D’Almeida J. (2005). Evaluation of repeated low energy impact damage in carbon-epoxy composite materials. Compos. Struct..

[5] Sadighi M., Alderliesten R. (2022). Impact fatigue, multiple and repeated low-velocity impacts on FRP composites: A review. Compos. Struct..

[6] Katunin A., Pawlak S., Wronkowicz-Katunin A., Tutajewicz D. (2020). Damage progression in fibre reinforced polymer composites subjected to low-velocity repeated impact loading. Compos. Struct..

[7] Zhou J., Wen P., Wang S. (2020). Numerical investigation on the repeated low-velocity impact behavior of composite laminates. Compos. Part B.

[8] Li L., Sun L., Wang T., Kang N., Cao W. (2019). Repeated low-velocity impact response and damage mechanism of glass fiber aluminium laminates. Aerosp. Sci. Technol..

[9] Guo K., Zhu L., Li Y., Yu T., Shenoi A., Zhou Q. (2018). Experimental investigation on the dynamic behaviour of aluminum foam sandwich plate under repeated impacts. Compos. Struct..

[10] Wang C., Chen Z., Silberschmidt V.V., Roy A. (2018). Damage accumulation in braided textiles-reinforced composites under repeated impacts: Experimental and numerical studies. Compos. Struct..

[11] Sevkat E., Liaw B., Delale F., Raju B.B. (2010). Effect of repeated impacts on the response of plain-woven hybrid composites. Compos. Part B.

[12] Atas C., Icten B.M., Küçük M. (2013). Thickness effect on repeated impact response of woven fabric composite plates. Compos. Part B.

[13] Zhou J., Liu B., Wang S. (2022). Finite element analysis on impact response and damage mechanism of composite laminates under single and repeated low-velocity impact. Aerosp. Sci. Technol..

[14] Dogan A. (2019). Single and repeated low-velocity impact response of E-glass fiber-reinforced epoxy and polypropylene composites for different impactor shapes. J. Thermoplast. Compos. Mater..

[15] Sun Z., Li C., Tie Y. (2021). Experimental and numerical investigations on damage accumulation and energy dissipation of patch-repaired CFRP laminates under repeated impacts. Mater. Des..

[16] Icten B.M. (2015). Low temperature effect on single and repeated impact behavior of woven glass-epoxy composite plates. J. Compos. Mater..

[17] Liao B., Zhou J., Li Y., Wang P., Xi L., Gao R., Bo K., Fang D. (2020). Damage accumulation mechanism of composite laminates subjected to repeated low velocity impacts. Int. J. Mech. Sci..

[18] Belingardi G., Cavatorta M.P., Paolino D.S. (2008). A new damage index to monitor the range of the penetration process in thick laminates. Compos. Sci. Technol..

